# Preventive effects of low-dose radiation and hypofractionated radiation plus anti-programmed cell death protein 1 on lung metastasis in breast cancer

**DOI:** 10.32604/or.2024.052133

**Published:** 2025-02-28

**Authors:** SHUANG CHEN, XUEMEI DENG, XINGTING HE, KEWEI XIANG, GUIHONG CHEN, HONGRU YANG

**Affiliations:** 1 Science and Technology Department, Southwest Medical University, Luzhou, 644600, China; 2 Department of Oncology, the Affiliated Hospital, Southwest Medical University, Luzhou, 644600, China

**Keywords:** Low-dose radiation therapy (LDRT), Hypo-fractionated radiation therapy (HFRT), Anti-programmed cell death protein 1 (αPD-1), Immune checkpoint inhibitors, Breast cancer

## Abstract

**Background:**

Previous experiments have demonstrated that hypofractionated radiation therapy (HFRT), low-dose radiation therapy (LDRT), and combined anti-programmed cell death protein 1 (αPD-1) can enhance the abscopal effect. Combined with the phenomenon of low prognosis in patients with breast cancer lung metastasis, our study establishes a mouse model and changes the irradiation regimen of LDRT to explore its preventive effect on breast cancer lung metastasis.

**Methods:**

The breast cancer subcutaneous graft tumor model was developed. Two-lung prophylactic LDRT was performed prior to the onset of lung metastases, in combination with HFRT (8 Gy, 3f), and αPD-1 (200 μg, 4f) therapy. We watched and documented the tumor volume, survival duration, and number of lung metastases. Furthermore, after labeling the corresponding cells using markers, we detected immune-related cell infiltration by immunohistochemistry and flow cytometry, such as T cells. We also determined the expression of cytokines (IFN-γ and TNF-α) by enzyme-linked immunosorbent assay.

**Result:**

The triple therapy (HFRT+LDRT+αPD-1) resulted in tumor shrinkage and prolonged survival in mice, with median survival extending from 35 to 52 days. The most notable decrease in the quantity of advanced lung metastatic nodules in breast cancer was observed with the triple therapy (HFRT+LDRT+αPD-1) (*p* < 0.05). Furthermore, according to immunohistochemistry and flow cytometry, the triple treatment (HFRT+LDRT+αPD-1) showed the greatest expression of CD8^+^ T cells. Additionally, the ratio of CD8^+^/CD4^+^ T cells was considerably greater than that of the groups (*p* < 0.0001). Triple therapy (HFRT+LDRT+αPD-1) increased the recruitment of DCs cells, promoted IFN-γ and TNF-α expression, and curbed the aggregation of MDSCs cells (*p* < 0.05).

**Conclusion:**

Prophylactic LDRT to the lungs, based on HFRT and αPD-1, can enhance anti-tumor efficacy and prevent advanced lung metastases from breast cancer. The process involves boosting the recruitment of DCs and CD8^+^ T cells, preventing MDSC cell aggregation, and lessening the tumor microenvironment’s immunosuppressive effects.

## Introduction

Hypofractionated radiation therapy (HFRT) activates innate and adaptive anti-tumor immunity through various mechanisms. It plays a pivotal role in regulating the tumor microenvironment (TME) and shifting the TME towards an immunologically favorable phenotype, producing local anti-tumor and abscopal effects [1,2]. Abscopal effects induced by HFRT alone are rare, and anti-programmed cell death protein 1 (αPD-1) plays an important role in this process [3–5]. However, HFRT is a double-edged sword, which can have negative effects by recruiting immunosuppressive cells and increasing the secretion of immunomodulatory cytokines [6–8].

Recent studies have shown that low-dose radiation therapy (LDRT) plays a key role in immunomodulation by enhancing the infiltration of immune effector cells [9–12]. This effect may attenuate the immunosuppressive effects of high-dose radiotherapy [13,14]. How to combine the different treatments to utilize their respective advantages and further expand the abscopal effect is a hot research topic in recent years [15–17]. For example, Yin et al. established a multi-tumor model to confirm that simultaneous HFRT (8 Gy, 3f, to the primary tumor), LDRT (2 Gy, 1f, to the secondary tumor), and immunotherapy significantly reduced the volume of metastatic tumors [16].

The highly metastatic nature of breast cancer, such as bone, lung, and liver metastases, has led to it being the malignant tumor with the highest mortality rate among women worldwide [18–20]. In recent years, researchers have made significant progress in breast cancer treatment, but the 5-year survival rate of patients with breast cancer lung metastasis is only 6.8% [21,22]. Based on the above research background, we wanted to change the irradiation regimen of LDRT, combine HFRT and αPD-1, to explore whether they could have a preventive effect on breast cancer lung metastasis.

## Materials and Methods

### Cell lines and tumor models

The mouse breast cancer cell line 4T1 (Bio-Tech Co., Shanghai, China) was acquired and cultivated in Dulbecco’s Modified Eagle Medium (BD Bioscience, San Diego, CA, USA), which included 1% penicillin/streptomycin (Beyotime, Shanghai, China) and 10% fetal bovine serum (BD Bioscience, USA). Female BALB/c mice, 6 weeks old (18 ± 2 g, HFK Bioscience, Beijing, China), were housed in specific pathogen-free environments 36 mice were randomly grouped before the experiment. BALB/c mice were subcutaneously (s.c.) inoculated with 1.5 × 10^5^ 4T1 cells in the right hind limb (the primary tumor). Digital calipers were used to measure tumors twice a week Tumor volume (mm^3^) = 0.5 × long diameter × short diameter^2^. This study was approved by the institutional animal care and use committee of Southwest Medical University. Ethical approval No. 20211021-001.

### Tumor therapy

The mice were given treatment when the original tumor size was between 60 and 80 mm^3^. Each mouse received isoflurane anesthesia (maintenance concentration: 1%–1.5%) prior to radiation therapy, and a lead box was used, such that only the tumor was exposed. The dose of irradiation for HFRT was 8 Gy, and irradiation was performed once a day for a total of 3 times. On the first day of HFRT irradiation, bilateral lung LDRT irradiation (0.1 Gy, 1f) was performed simultaneously. αPD-1 was administered intraperitoneally (200 µg per injection; BP0273, Bio X Cell, West Lebanon, NH, USA) every 3 days for four treatments. When the main tumor reached a volume of 2000 mm^3^, the mice were put to death in accordance with ethical animal protection rules. Survival was calculated from the date of tumor cell implantation to the date of death.

### Metastatic nodule count

The mice were killed at defined experimental endpoints, their lungs were collected, and metastatic nodules were counted under a dissecting microscope. Grading was performed according to the diameter, where <0.5 mm was defined as grade A, 0.5–1 mm was defined as grade B, 1–2 mm was defined as grade C, and >2 mm was defined as grade D. The total number of lesions was equal to the sum of A × 1, B × 2, C × 3, and D × 4 [23].

### Flow cytometry

After anesthesia, mice were executed, and tumor and spleen tissues were obtained. Tumor tissues were minced into pulp, mixed with 3 mL of digestive enzyme, and incubated for 30 min at 37°C. Spleen tissues were ground to pulp, mixed with an equal amount of erythrocyte lysate, and incubated for 20 min at 37°C. Aliquots of medium were added to the tissues to terminate the digestion, and then the tissues were filtered through a 70 μm pore-size nylon gauze. We gathered the last single-cell suspensions. Subsequently, the cell suspensions were incubated with fluorescently labeled antibodies (BD Bioscience, USA) against CD3 (553061), CD4 (551162), CD8 (553128), CD11b (553079), CD11c (550993), MHC II (553051), CD45 (550261), and Gr1 (557000) at 37°C for 30 min. A FACSAria flow cytometer (BD Bioscience, USA) was used for the multicolor flow cytometry study. Treestar Inc., Ashland, OR, USA, provided the Flow Jo program, which was used to further analyze the data.

### Liquid chip assay and enzyme-linked immunosorbent assay (ELISA)

Sections of pulmonary metastatic nodules were incubated with CD3, CD4, and CD8 antibodies for 1 h at 37°C after immunohistochemistry-related pretreatment. Subsequently, the sections were incubated with anti-mouse secondary antibody for 1 h at 37°C and stained dropwise with DAB reagent (Beyotime, China) for 5–10 min. Image acquisition was performed using an inverted fluorescence microscope after blocking the sections. Media Cybernetics, Image-Pro Plus 6.0 software was used for the quantitative analysis. The concentration of cytokines (IFN-γ and TNF-α) in mouse serum was measured using a mouse-ELISA kit (BD Bioscience, USA) according to the manufacturer’s protocol.

### HE staining

Lung tissue sections were stained by immersion in hematoxylin solution for 5 min (if reverse blue, alkaline buffer solution could be used), and the surface water was blotted out after soaking in tap water for 15 min. Sections were immersed in 5% acetic acid for 30s, soaked in tap water for 15 min, and then drained again. Sections were immersed in eosin solution for 1 min and rinsed in tap water for 1 min. Sections were sequentially immersed in 100%, 95%, 70%, and 50% ethanol for 10 min for dehydration, and then immersed in xylene solution for 10 min for clearing. Finally, the sections were dropwise added with neutral gum, and coverslips were used to seal the sections. The morphological structure was observed under a light microscope and pictures were collected.

### Statistical analysis

All statistical analyses were performed using GraphPad Prism 7.0 (La Jolla, CA, USA). The mean ± standard error of the mean is used to represent the findings. *t*-tests for students were used to assess the significance among the groups. The Kaplan-Meier technique was utilized to examine the survival percentages, and log-rank tests were employed for comparison. A two-way analysis of variance (ANOVA) was used where applicable to compare tumor growth curves. *p* < 0.05, statistical significance was established.

## Results

### Triple therapy synergistically enhances tumor control and overall survival in mice

**Figure 1:**
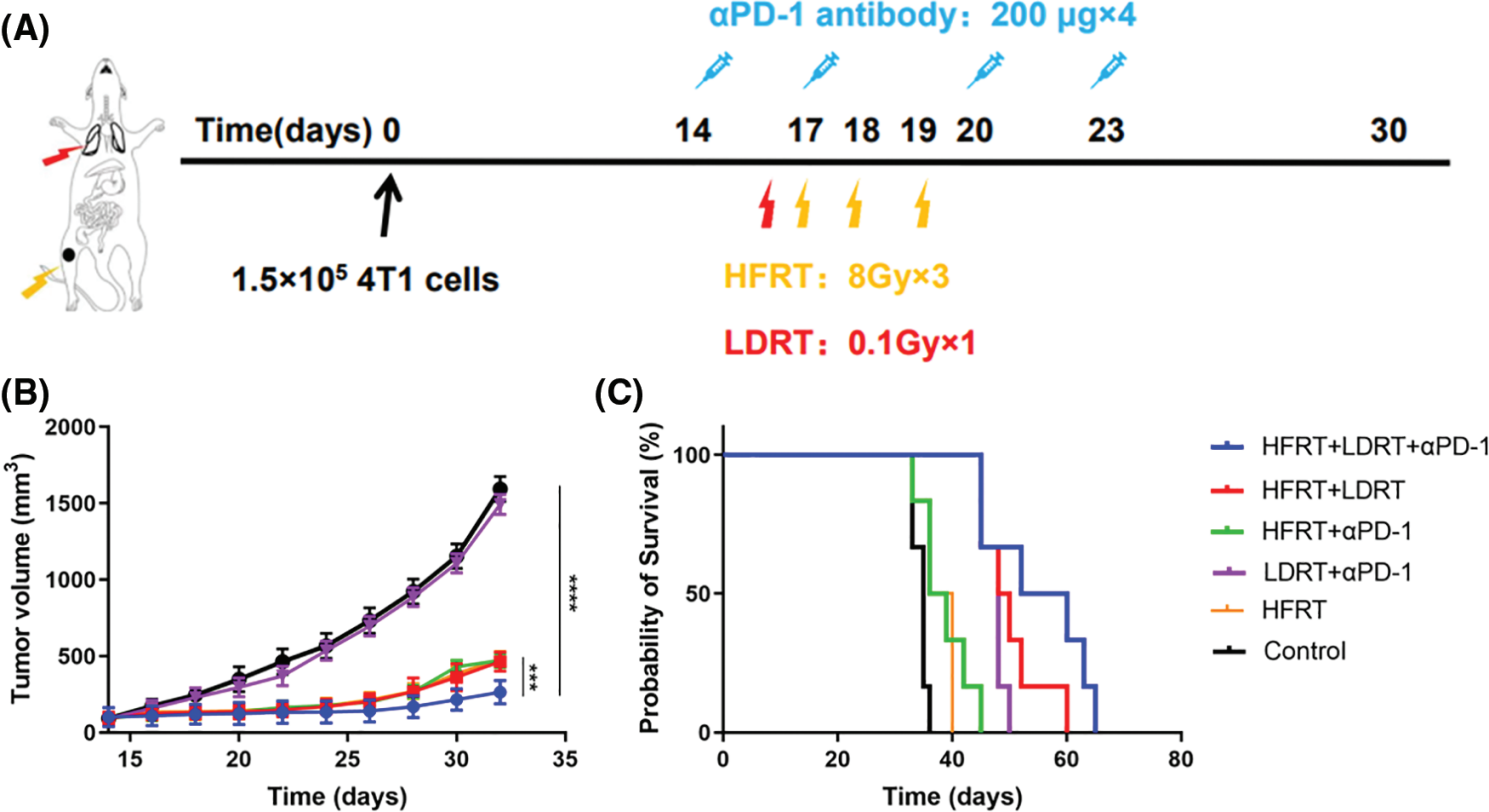
Systemic synergistic effects of triple therapy. (A) Treatment scheme; (B) Tumor growth curve (*n* = 6); (C) Mouse survival curve (*n* = 6). ***p < 0.001, ****p < 0.0001.

In this study, mice bearing a single 4T1 tumor were used. Fig. 1A illustrates the therapy protocol schematically. With the exception of the control and LDRT+αPD-1 groups, the growth of subcutaneous graft tumors was significantly slowed, with the strongest decrease observed in the triple therapy group (Fig. 1B). Prolonged survival was observed in mice treated with triple therapy, which extended the median survival from days 35 to 52, while HFRT+LDRT and LDRT+αPD-1 extended the median survival to day 48, and HFRT+αPD-1 and HFRT extended the median survival to day 36 (Fig. 1C).

### Triple therapy prevents the development of lung metastases in advanced breast cancer

**Figure 2:**
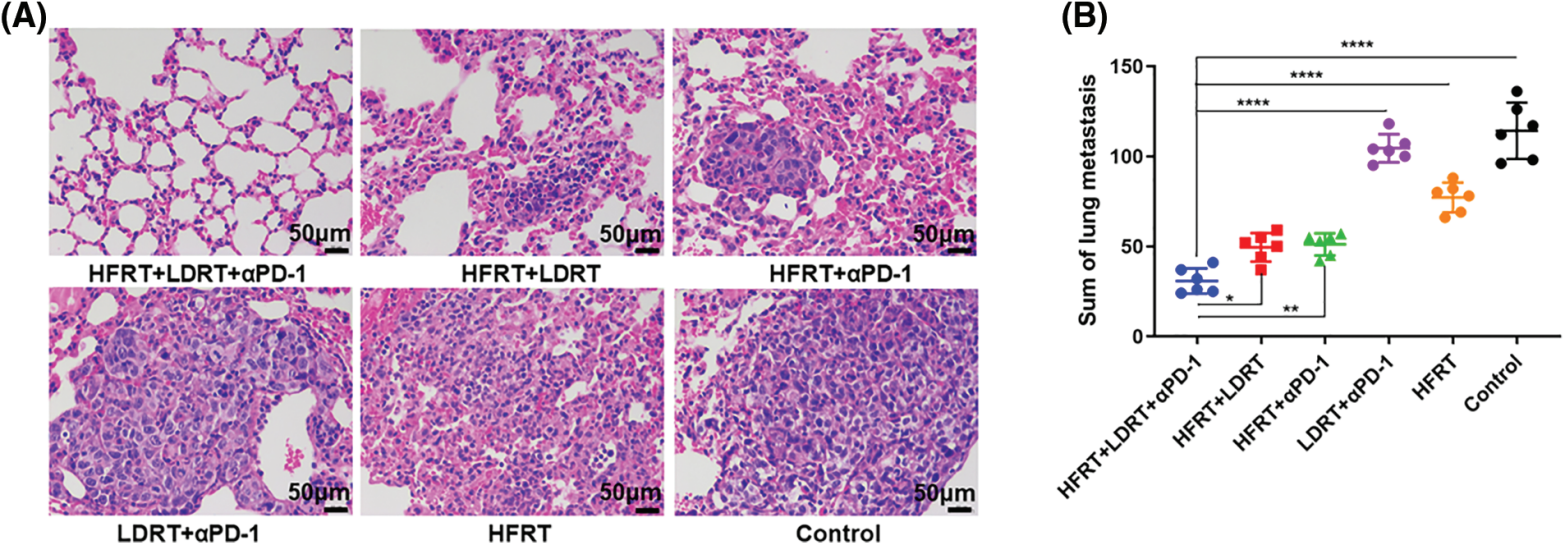
The tumor metastasis preventive role of triple therapy. (A) H&E-stained of lung (original magnification ×400); (B) Number of lung metastatic nodules. *p < 0.05, **p < 0.01, ****p < 0.0001.

LDRT was performed in parallel with HFRT in the early stages of treatment, with the aim to modulate the stroma in advance and allow effector immune cells to infiltrate/expand and exert a preventive effect on lung metastasis (Fig. 2). As expected, we found that delivering HFRT to primary tumors with LDRT to bilateral lungs and systemic αPD-1 noticeably enhanced systemic abscopal responses and significantly reduced metastatic nodules (Fig. 2B). However, LDRT+αPD-1 did not prevent lung metastases in mice with advanced breast cancer. Moreover, no larger tumor masses were observed in the H&E-stained mouse lung tissues treated with triple therapy (Fig. 2A).

### Triple therapy modifies effector Tlymphocyte expression in the local and systemic tumor immune microenvironment

**Figure 3:**
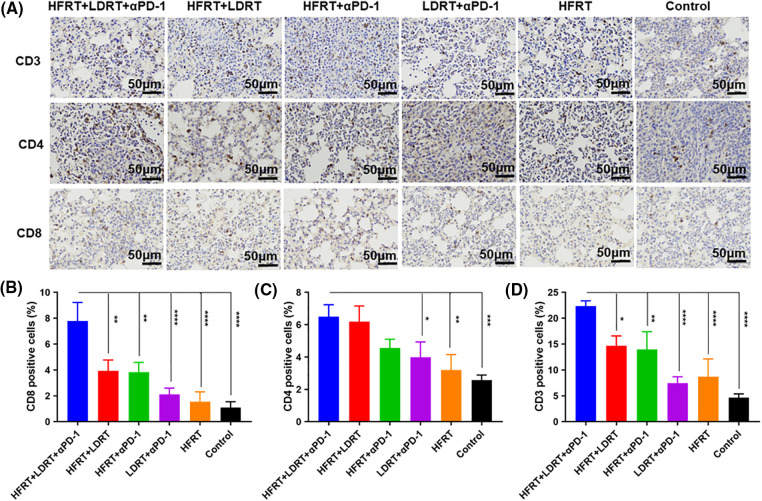
Effector T cell infiltration in lung metastatic nodal tissues. (A) IHC staining (the lung metastatic nodal tissues’ CD3^+^/CD4^+^/CD8^+^ cells are represented by the brown dots, original magnification ×400); (B) Percentages of CD3^+^ cells; (C) Percentages of CD4^+^ cells; (D) Percentages of CD8^+^ cells. *p < 0.05, **p < 0.01, ***p < 0.001, ****p < 0.0001.

**Figure 4:**
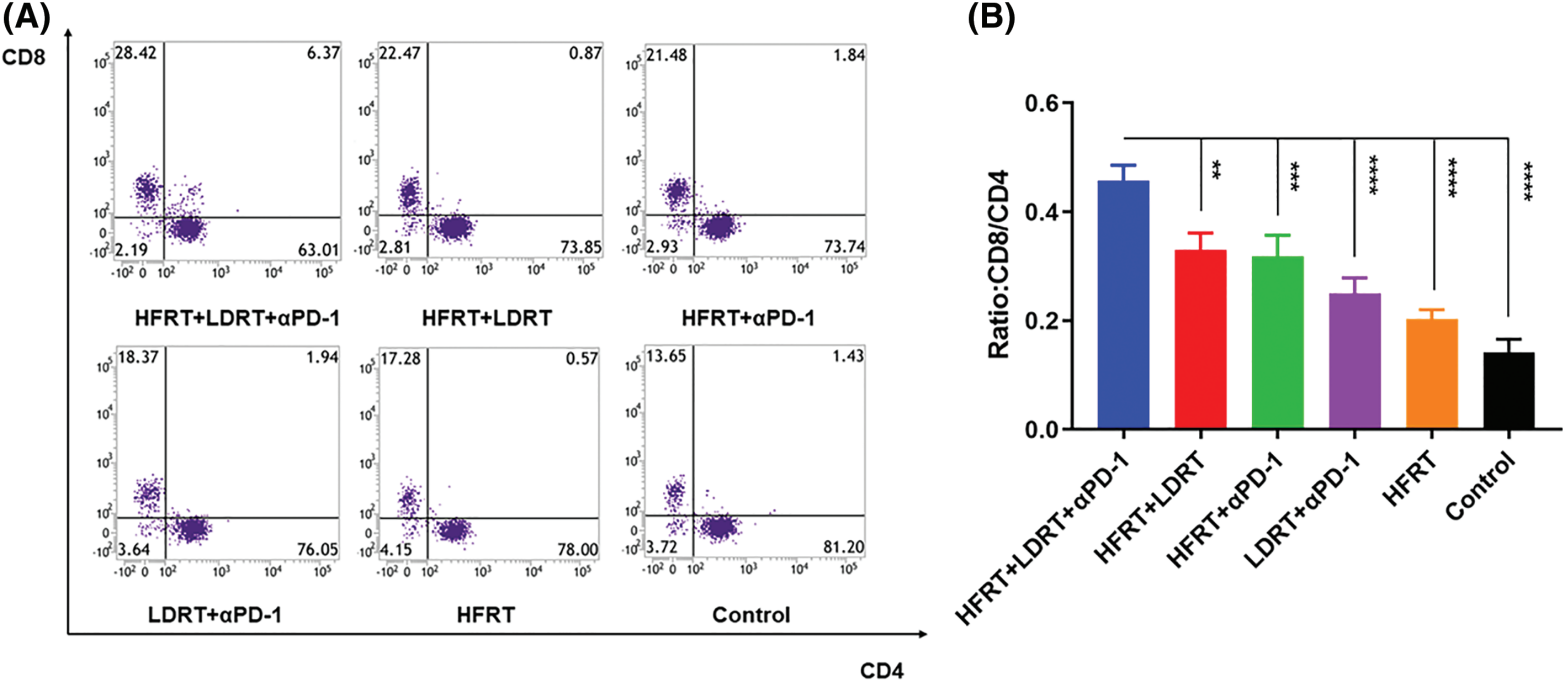
Effector T cell infiltration in spleen tissues. (A) Frequencies of CD3^+^CD8^+^ T cells and CD3^+^CD4^+^ T cells; (B) Ratio of CD8^+^ T cells to CD4^+^ T cells. **p <0.01, ***p < 0.001, ****p < 0.0001.

To explore the effect of triple therapy on immune cell subsets, we obtained lung metastatic nodal tissues from tumor-bearing mice and stained them with the T cell markers CD3, CD4, and CD8 (Fig. 3A). We found that all of the protocols induced the infiltration of CD3^+^, CD4^+^, and CD8^+^ T cells, with the strongest increase observed in response to triple therapy (Fig. 3B–D). However, as immunohistochemistry has certain limitations when it comes to labeling these two markers concurrently and cytotoxic T cells are often described as CD3^+^CD8^+^ T cells, we used flow cytometry to assess the expression ratio of CD4^+^CD8^+^ T cells in the spleen (Fig. 4A). The experiment showed that the triple therapy group had the highest level of CD8^+^ T cell expression in the spleen and had a considerably higher CD8^+^/CD4^+^ T cell ratio than the other groups (*p* < 0.0001, Fig. 4B).

### Triple therapy impacts DC and MDSC expression in the systemic tumor immune microenvironment

**Figure 5:**
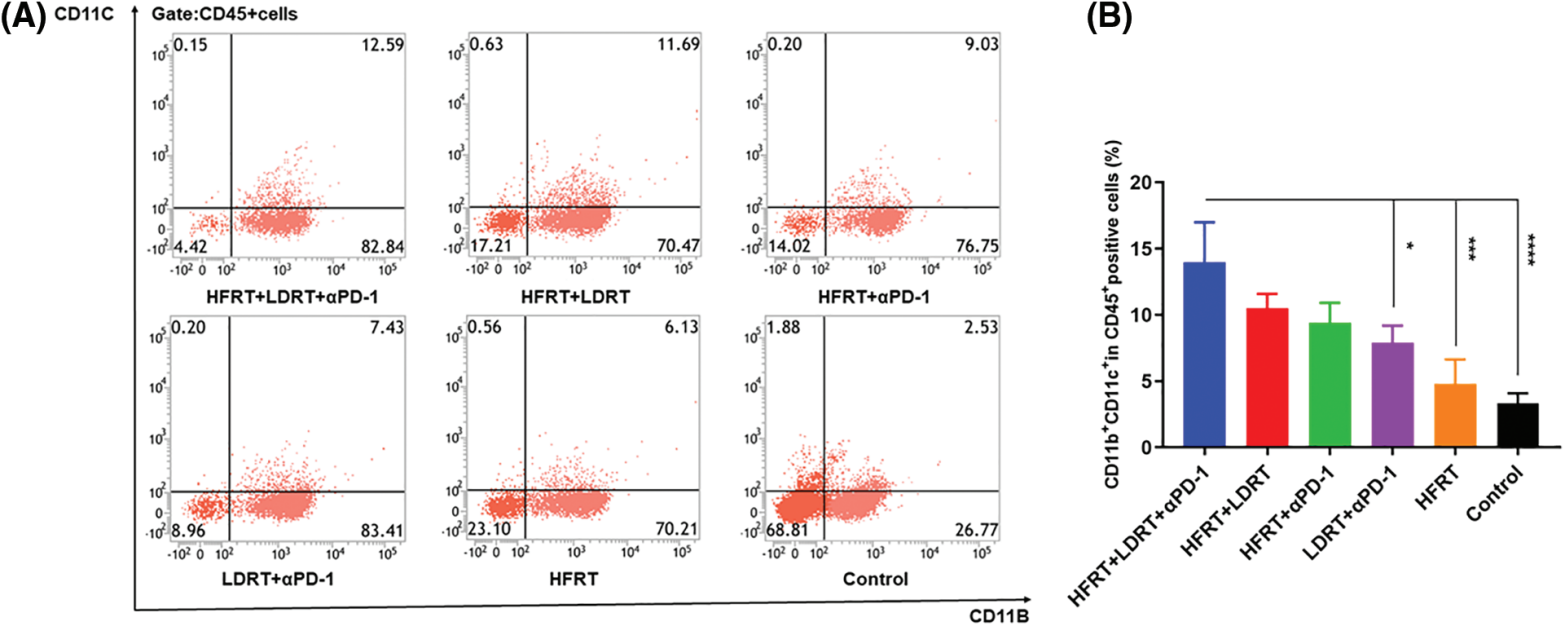
Infiltration of DCs into spleen tissues. (A) Frequencies of CD45^+^CD11b^+^CD11c^+^ cells; (B) Percentage of CD45^+^CD11b^+^CD11c^+^ cells. *p < 0.05, ***p < 0.001, ****p < 0.0001.

We next quantified the proportion of tumor-associated DCs in splenic tissue by gating on CD45^+^CD11b^+^CD11c^+^ populations (Fig. 5A). Comparing the triple therapy group to the HFRT (*p* < 0.001), LDRT+αPD-1 (*p* < 0.05), and control (*p* < 0.0001) groups, the triple therapy group had a higher percentage of CD45^+^CD11b^+^CD11c^+^ cells (Fig. 5B).

**Figure 6:**
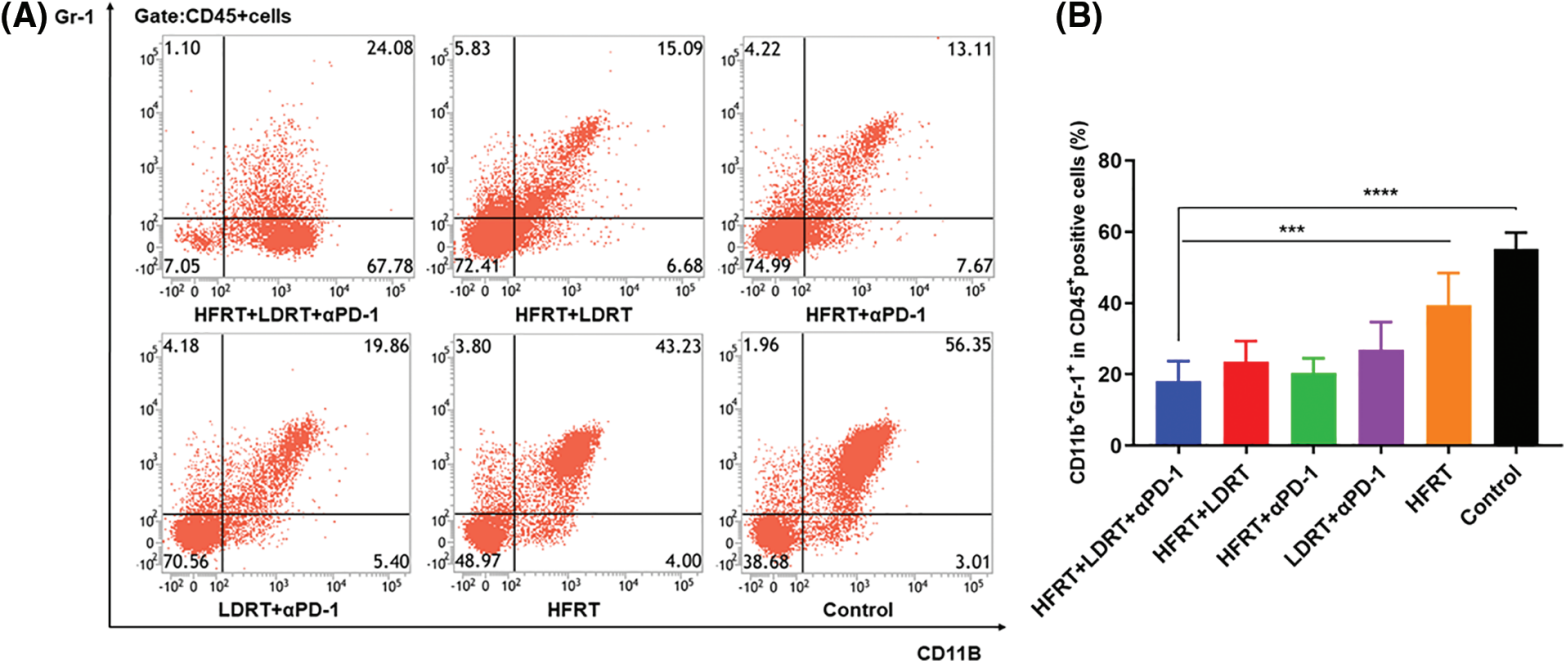
Infiltration of MDSCs into spleen tissues. (A) Frequencies of CD45^+^CD11b^+^Gr-1^+^ cells; (B) Percentages of CD45^+^CD11b^+^Gr-1^+^ cells. ***p < 0.001, ****p < 0.0001.

Meanwhile, we extracted splenic tissue and used gating on CD45^+^CD11b^+^Gr-1^+^ populations to analyze the impact of various treatment regimens on the MDSC population (Fig. 6A). Additionally, there was little variation in the percentage of CD45^+^CD11b^+^Gr-1^+^ cells in the HFRT+LDRT, HFRT+αPD-1, and LDRT+αPD-1 groups compared with those in the triple therapy group (Fig. 6B). On the other hand, the triple therapy group’s percentage of CD45^+^CD11b^+^Gr-1^+^ cells significantly decreased compared with those in the HFRT (*p* < 0.001) and control (*p* < 0.0001) groups (Fig. 6B). Overall, triple therapy showed increased infiltration of DCs and decreased infiltration of MDSCs. This reduced the immunosuppressive character of the tumor microenvironment (TME) and created a milieu that was conducive to T cells mounting an anticancer immune response.

### Triple therapy affects cytokine expression in the systemic tumor immune microenvironment

**Figure 7:**
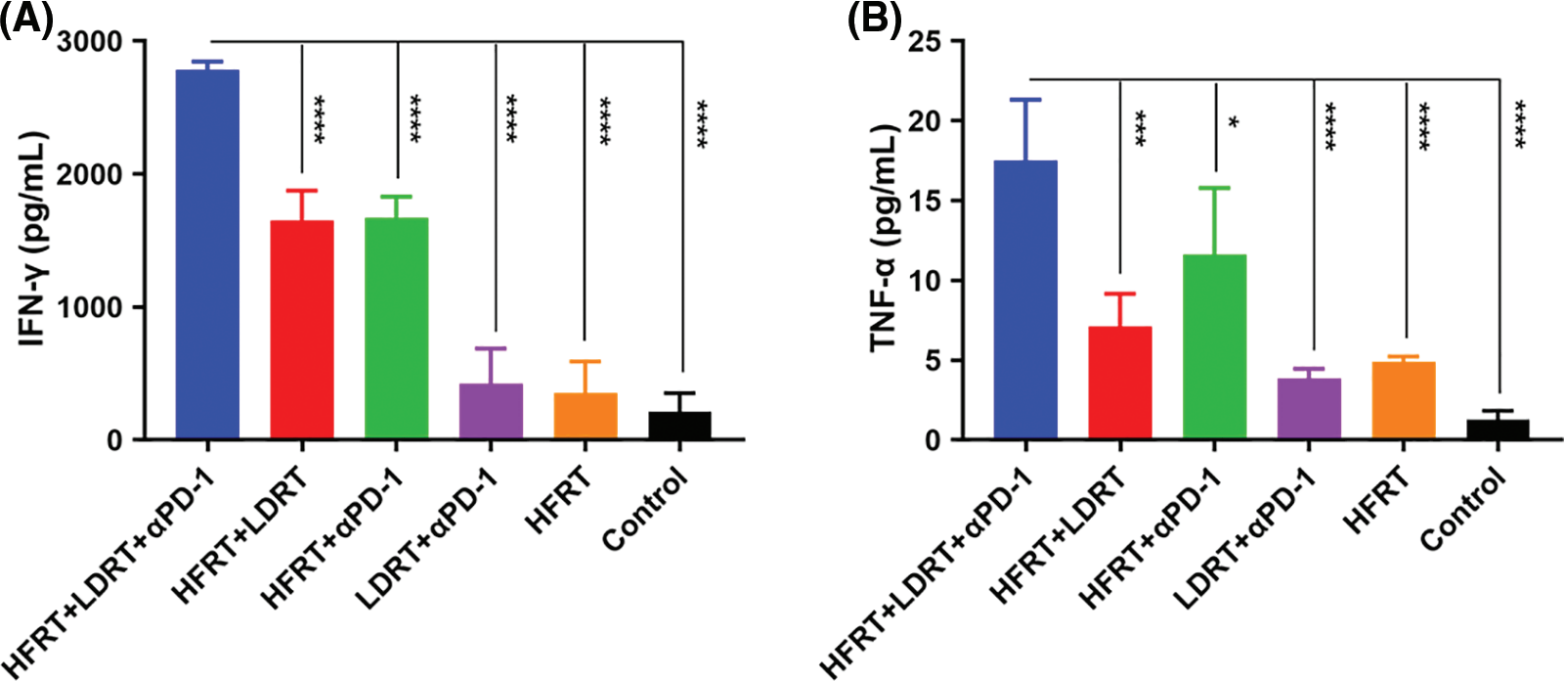
Expression of serum cytokines. (A) ELISA results in IFN-γ (pg/mL); (B) ELISA results in TNF-α levels (pg/mL). *p < 0.05, ***p < 0.001, ****p < 0.0001.

We next determined the serum levels of IFN-γ and TNF-α, given their potential involvement in the tumor control effect mechanism of triple therapy. On day 32, the combination treatment groups—especially the triple therapy group—had greater blood levels of TNF-α and IFN-γ (Fig. 7). Specifically, the triple therapy group showed approximately higher levels of IFN-γ (1.7-fold, Fig. 7A) and TNF-α (1.5-fold, Fig. 7B) as compared to the HFRT+αPD-1 group, indicating that LDRT may be the key driver elevating serum levels of IFN-γ and TNF-α.

## Discussion

The extent to which RT stimulates the immune system and induces antitumor immune effects is variable and depends on the type of cancer, RT dose, RT fractionation, and many other factors [24,25]. The low incidence of abscopal effects of RT combined with immunotherapy suggests that optimal biological effects are still not achieved despite the release of tumor antigens and activation of antigen-presenting cells and effector T cells by high-dose radiation therapy. Recent studies have shown that low-dose radiation therapy can further enhance the *in situ* inoculation effect of high-dose radiation therapy by promoting APC maturation (CD40 agonists), enhancing effector T-cell infiltration (CTLA-4 blockers), and attenuating immunosuppressive signals (TGF-β, Tregs), which can play complementary roles [26–29].

We believe that the administration of high-dose radiation to the primary tumor site can be used to induce tumor neoantigens, while low-dose radiation administered to the lungs to reprogram the TME, combined with immunotherapy, may maximize anti-tumor immunity. This is also illustrated by the fact that the triple therapy group had the strongest effect in delaying tumor growth in the results of this experiment. The outcomes line up with Yin et al.’s analysis, who established a double-tumor mouse model of lung cancer for HFRT (8 Gy, 3f) for the primary tumor and LDRT (2 Gy) for the secondary tumor combined with immunotherapy [16].

Abscopal effects have been demonstrated in melanoma, renal cell carcinoma, lymphoma, lung cancer, and others, with an overall “random” character [2–5]. Inhibition of secondary tumor growth is only one manifestation of the abscopal effect, while inhibition of tumor metastasis (e.g., pulmonary metastasis) is another. Indeed, many investigators have observed a decrease in tumor metastasis in trials in which radiation therapy was combined with other treatments. Wang et al. observed a significant reduction in the incidence of lung metastases when high-dose radiotherapy (12 Gy, 3f) combined with immunotherapy was administered to subcutaneous transplanted tumors of lung cancer [15]. Savage et al. observed a significant reduction in spontaneous pulmonary metastatic nodules after high-dose radiotherapy (20 Gy, 3f) followed by LDRT (0.5 Gy, 4f) [14].

The reduced number of pulmonary metastatic nodules in the HFRT group demonstrated that HFRT (8 Gy, 3f) could exert anti-tumor effects outside the irradiated area, producing an abscopal effect, in line with the results of previous studies [16,17]. This effect was further enhanced by combining immunotherapy with low-dose radiotherapy, as seen in our experimental results. Among them, the reduction in the number of lung metastatic nodules was most significant in the HFRT+LDRT+αPD-1 group, and no larger tumor masses were observed by HE staining, indicating that the effects of immunotherapy and low-dose radiotherapy enhanced by HFRT can be superimposed on each other. Unlike Wang et al., who established a mouse model of lung cancer and performed HFRT (12 Gy, 3f) combined with immunotherapy [15], we established a mouse model of 4T1 breast cancer with a high metastatic cell line and added LDRT (0.1 Gy, 1f) in the lungs to HFRT (8 Gy, 3f) combined with immunotherapy. Savage et al. also established a mouse model of breast cancer and performed HFRT (20 Gy, 3f) combined with LDRT (0.5 Gy, 4f), but not combined with immunotherapy [14], which differed from our experiment. Unlike the experiment of Liu et al. [25], only low-dose radiotherapy to the lungs was performed in this experiment because the cell lines we chose are very susceptible to lung metastasis. We hope that this treatment modality can be extended to clinical application after validation in the future and that narrowing the irradiation area can reduce the occurrence of adverse effects of radiotherapy.

The entire process of tumor development is undoubtedly accompanied by changes in the tumor microenvironment [30–33]. Radiation therapy induces the production of death receptors in tumor cells, which act by binding to activated T lymphocytes, such as TNF-α and TRAIL [34–37], to induce the secretion of chemokines by different types of tumor cells. Consequently, these secreted chemokines, such as CXCL9 and CXCL10 [38–41], recruit effector CD8^+^ T cells and helper CD4^+^ T cells, which in turn produce an abscopal effect. Thus, the abscopal effect is inextricably linked to T cells. Indeed, in the current study, the CD8^+^/CD4^+^ T-cell ratios in the spleen tissues of the groups were significantly different (Figs. 3 and 4), indicating that the induced stimulatory effect of triple therapy on T cells was mainly reflected in increasing the number of infiltrating CD8^+^ T cells, but not CD4^+^ T cells. Furthermore, CD8^+^ T-cell infiltration was increased the lung metastasis tissue in the triple therapy group compared with the other groups, consistent with the findings of previous studies.

In summary, early administration of prophylactic LDRT to the lungs, based on HFRT and αPD-1 monoclonal antibody, successfully delayed the growth of subcutaneous transplanted tumors in 4T1 breast cancer, prolonged the survival of tumor-bearing mice, and reduced the number of advanced spontaneous lung metastases. These results suggest that early prophylactic LDRT in both lungs may prevent spontaneous lung metastases from breast cancer, a phenomenon that is associated with the infiltration of CD8^+^ T cells, DCs, and MDSC to alter the tumor microenvironment. However, this conclusion needs to be validated in another tumor model.

There are some shortcomings in our experiments. First, we selected high metastatic 4T1 breast cancer cells to establish an animal tumor model. We did not select other breast cancer cell lines to establish tumor models for validation. Second, the tumor microenvironment is rich in the variety of immune cells and cytokines associated with anti-tumor effects, and we did not perform comprehensive testing. The clear mechanism of combination therapy and its complex signaling pathways are still unclear. In the future, we will make adjustments to address the above issues and improve the enrichment experiments in order to further validate our results.

## Conclusions

Our results demonstrate that prophylactic LDRT to the lungs, based on HFRT and αPD-1, can enhance anti-tumor efficacy and prevent advanced lung metastases from breast cancer. The process involves boosting the recruitment of DCs and CD8^+^ T cells, preventing MDSC cell aggregation, and lessening the tumor microenvironment’s immunosuppressive effects.

## Data Availability

All data generated or analyzed during this study are included in this published article.

## References

[1] Frey B, Borgmann K, Jost T, Greve B, Oertel M, Micke O, et al. DNA as the main target in radiotherapy-a historical overview from first isolation to anti-tumour immune response. Strahlenther Onkol. 2023;199(12):1080–90. doi:10.1007/s00066-023-02122-5; 37620671

[2] Barsoumian HB, Ramapriyan R, Younes AI, Caetano MS, Menon H, Comeaux NI, et al. Low-dose radiation treatment enhances systemic antitumor immune responses by overcoming the inhibitory stroma. J Immunother Cancer. 2020;8(2):e000537. doi:10.1136/jitc-2020-000537; 33106386 PMC7592253

[3] Kodet O, Němejcova K, Strnadová K, Havlínová A, Dundr P, Krajsová I, et al. The abscopal effect in the era of checkpoint inhibitors. Int J Mol Sci. 2021;22(13):7204. doi:10.3390/ijms22137204; 34281259 PMC8267720

[4] Formenti SC, Rudqvist NP, Golden E, Cooper B, Wennerberg E, Lhuillier C, et al. Radiotherapy induces responses of lung cancer to CTLA-4 blockade. Nat Med. 2018;24(12):1845–51. doi:10.1038/s41591-018-0232-2; 30397353 PMC6286242

[5] Uryvaev A, Passhak M, Hershkovits D, Sabo E, Bar-Sela G. The role of tumor-infiltrating lymphocytes (TILs) as a predictive biomarker of response to anti-PD1 therapy in patients with metastatic non-small cell lung cancer or metastatic melanoma. Med Oncol. 2018;35(3):25. doi:10.1007/s12032-018-1080-0; 29388007

[6] Chen Y, Gao M, Huang Z, Yu J, Meng X. SBRT combined with PD-1/PD-L1 inhibitors in NSCLC treatment: a focus on the mechanisms, advances, and future challenges. J Hematol Oncol. 2020;13(1):105. doi:10.1186/s13045-020-00940-z; 32723363 PMC7390199

[7] Chen D, Barsoumian HB, Fischer G, Yang L, Verma V, Younes AI, et al. Combination treatment with radiotherapy and a novel oxidative phosphorylation inhibitor overcomes PD-1 resistance and enhances antitumor immunity. J Immunother Cancer. 2020;8(1):e000289. doi:10.1136/jitc-2019-000289; 32581056 PMC7319777

[8] Lin L, Kane N, Kobayashi N, Kono EA, Yamashiro JM, Nickols NG, et al. High-dose per fraction radiotherapy induces both antitumor immunity and immunosuppressive responses in prostate tumors. Clin Cancer Res. 2021;27(5):1505–15. doi:10.1158/1078-0432.CCR-20-2293; 33219015

[9] Herrera FG, Ronet C, De Olza MO, Barras D, Crespo I, Andreatta M, et al. Low-dose radiotherapy reverses tumor immune desertification and resistance to immunotherapy. Cancer Discov. 2022;12(1):108–33. doi:10.1158/2159-8290.CD-21-0003; 34479871 PMC9401506

[10] Zhang QF, Li J, Jiang K, Wang R, Ge JL, Yang H, et al. CDK4/6 inhibition promotes immune infiltration in ovarian cancer and synergizes with PD-1 blockade in a B cell-dependent manner. Theranostics. 2020;10(23):10619–33. doi:10.7150/thno.44871; 32929370 PMC7482823

[11] Monjazeb AM, Giobbie-Hurder A, Lako A, Thrash EM, Brennick RC, Kao KZ, et al. A randomized trial of combined PD-L1 and CTLA-4 inhibition with targeted low-dose or hypofractionated radiation for patients with metastatic colorectal cancer. Clin Cancer Res. 2021;27(9):2470–80. doi:10.1158/1078-0432.CCR-20-4632; 33568343 PMC8102320

[12] Herrera FG, Bourhis J, Coukos G. Radiotherapy combination opportunities leveraging immunity for the next oncology practice. CA Cancer J Clin. 2017;67(1):65–85. doi:10.3322/caac.21358; 27570942

[13] Grass GD, Krishna N, Kim S. The immune mechanisms of abscopal effect in radiation therapy. Curr Probl Cancer. 2016;40(1):10–24. doi:10.1016/j.currproblcancer.2015.10.003; 26612692

[14] Savage T, Pandey S, Guha C. Postablation modulation after single high-dose radiation therapy improves tumor control via enhanced immunomodulation. Clin Cancer Res. 2020;26(4):910–21. doi:10.1158/1078-0432.CCR-18-3518; 31757878

[15] Wang X, Schoenhals JE, Li A, Valdecanas DR, Ye H, Zang F, et al. Suppression of type I IFN signaling in tumors mediates resistance to anti-PD-1 treatment that can be overcome by radiotherapy. Cancer Res. 2017;77(4):839–50. doi:10.1158/0008-5472.CAN-15-3142; 27821490 PMC5875182

[16] Yin L, Xue J, Li R, Zhou L, Deng L, Chen L, et al. Effect of low-dose radiation therapy on abscopal responses to hypofractionated radiation therapy and anti-PD1 in mice and patients with non-small cell lung cancer. Int J Radiat Oncol Biol Phys. 2020;108(1):212–24; 32417411 10.1016/j.ijrobp.2020.05.002

[17] Caetano MS, Younes AI, Barsoumian HB, Quigley M, Menon H, Gao C, et al. Triple therapy with MerTK and PD1 inhibition plus radiotherapy promotes abscopal antitumor immune responses. Clin Cancer Res. 2019;25(24):7576–84; 31540976 10.1158/1078-0432.CCR-19-0795 PMC6911635

[18] Sung H, Ferlay J, Siegel RL, Laversanne M, Soerjomataram I, Jemal A, et al. Global cancer statistics 2020: GLOBOCAN estimates of incidence and mortality worldwide for 36 cancers in 185 countries. CA Cancer J Clin. 2021;71(3):209–49; 33538338 10.3322/caac.21660

[19] Riggio AI, Varley KE, Welm AL. The lingering mysteries of metastatic recurrence in breast cancer. Br J Cancer. 2021;124(1):13–26; 33239679 10.1038/s41416-020-01161-4 PMC7782773

[20] Liang Y, Zhang H, Song X, Yang Q. Metastatic heterogeneity of breast cancer: molecular mechanism and potential therapeutic targets. Semin Cancer Biol. 2020;60:14–27; 31421262 10.1016/j.semcancer.2019.08.012

[21] Bradley JA, Mendenhall NP. Novel radiotherapy techniques for breast cancer. Annu Rev Med. 2018;69(1):277–88. doi:10.1146/annurev-med-042716-103422; 29195057

[22] Ignatov A, Eggemann H, Burger E, Ignatov T. Patterns of breast cancer relapse in accordance to biological subtype. J Cancer Res Clin Oncol. 2018;144(7):1347–55. doi:10.1007/s00432-018-2644-2; 29675790 PMC11813410

[23] Liu J, Zhou J, Wu M, Hu C, Yang J, Li D, et al. Low-dose total body irradiation can enhance systemic immune related response induced by hypo-fractionated radiation. Front Immunol. 2019;10:317. doi:10.3389/fimmu.2019.00317; 30873170 PMC6401363

[24] Solomon E, Lemberskiy G, Baete S, Hu K, Malyarenko D, Swanson S, et al. Time-dependent diffusivity and kurtosis in phantoms and patients with head and neck cancer. Magn Reson Med. 2023;89(2):522–35. doi:10.1002/mrm.29457; 36219464 PMC9712275

[25] Liu S, Liao Y, Chen Y, Yang H, Hu Y, Chen Z, et al. Effect of triple therapy with low-dose total body irradiation and hypo-fractionated radiation plus anti-programmed cell death protein 1 blockade on abscopal antitumor immune responses in breast cancer. Int Immunopharmacol. 2023;117(8):110026. doi:10.1016/j.intimp.2023.110026; 36934673

[26] Paganetti H. A review on lymphocyte radiosensitivity and its impact on radiotherapy. Front Oncol. 2023;13:1201500. doi:10.3389/fonc.2023.1201500; 37601664 PMC10435323

[27] Klug F, Prakash H, Huber PE, Seibel T, Bender N, Halama N, et al. Low-dose irradiation programs macrophage differentiation to an iNOS^+^/M1 phenotype that orchestrates effective T cell immunotherapy. Cancer Cell. 2013;24(5):589–602. doi:10.1016/j.ccr.2013.09.014; 24209604

[28] Liao Y, Chen Y, Liu S, Wang W, Fu S, Wu J. Low-dose total body irradiation enhances systemic anti-tumor immunity induced by local cryotherapy. J Cancer Res Clin Oncol. 2023;149(12):10053–63. doi:10.1007/s00432-023-04928-3; 37261526 PMC11796646

[29] Nadella V, Singh S, Jain A, Jain M, Vasquez KM, Sharma A, et al. Low dose radiation primed iNOS + M1macrophages modulate angiogenic programming of tumor derived endothelium. Mol Carcinog. 2018;57(11):1664–71. doi:10.1002/mc.22879; 30035346 PMC7051861

[30] Wang S, Campos J, Gallotta M, Gong M, Crain C, Naik E, et al. Intratumoral injection of a CpG oligonucleotide reverts resistance to PD-1 blockade by expanding multifunctional CD8^+^ T cells. Proc Natl Acad Sci U S A. 2016;113(46):E7240–9. doi:10.1073/pnas.1608555113; 27799536 PMC5135381

[31] Wang D, Jiang W, Zhu F, Mao X, Agrawal S. Modulation of the tumor microenvironment by intratumoral administration of IMO-2125, a novel TLR9 agonist, for cancer immunotherapy. Int J Oncol. 2018;53(3):1193–1203. doi:10.3892/ijo.2018.4456; 29956749

[32] Welsh J, Menon H, Chen D, Verma V, Tang C, Altan M, et al. Pembrolizumab with or without radiation therapy for metastatic non-small cell lung cancer: a randomized phase I/II trial. J Immunother Cancer. 2020;8(2):e001001. doi:10.1136/jitc-2020-001001; 33051340 PMC7555111

[33] Myers CJ, Lu B. Decreased survival after combining thoracic irradiation and an anti-PD-1 antibody correlated with increased T-cell infiltration into cardiac and lung tissues. Int J Radiat Oncol Biol Phys. 2017;99(5):1129–36. doi:10.1016/j.ijrobp.2017.06.2452; 29165283 PMC5726785

[34] Theelen WSME, Chen D, Verma V, Hobbs BP, Peulen HMU, Aerts JGJV, et al. Pembrolizumab with or without radiotherapy for metastatic non-small-cell lung cancer: a pooled analysis of two randomised trials. Lancet Respir Med. 2021;9(5):467–75. doi:10.1016/S2213-2600(20)30391-X; 33096027

[35] Zhang X, Niedermann G. Abscopal effects with hypofractionated schedules extending into the effector phase of the tumor-specific T-cell response. Int J Radiat Oncol Biol Phys. 2018;101(1):63–73. doi:10.1016/j.ijrobp.2018.01.094; 29534901

[36] Turley SJ, Cremasco V, Astarita JL. Immunological hallmarks of stromal cells in the tumour microenvironment. Nat Rev Immunol. 2015;15(11):669–82; 26471778 10.1038/nri3902

[37] Rodriguez-Ruiz ME, Rodriguez I, Garasa S, Barbes B, Solorzano JL, Perez-Gracia JL, et al. Abscopal effects of radiotherapy are enhanced by combined immunostimulatory mAbs and are dependent on CD8 T cells and crosspriming. Cancer Res. 2016;76(20):5994–6005; 27550452 10.1158/0008-5472.CAN-16-0549

[38] Rodríguez-Ruiz ME, Rodríguez I, Mayorga L, Labiano T, Barbes B, Etxeberria I, et al. TGFβ blockade enhances radiotherapy abscopal efficacy effects in combination with anti-PD1 and anti-CD137 immunostimulatory monoclonal antibodies. Mol Cancer Ther. 2019;18(3):621–31. 30683810 10.1158/1535-7163.MCT-18-0558

[39] De Cicco P, Ercolano G, Ianaro A. The new era of cancer immunotherapy: targeting myeloid-derived suppressor cells to overcome immune evasion. Front Immunol. 2020;11:1680; 32849585 10.3389/fimmu.2020.01680 PMC7406792

[40] Gao X, Sui H, Zhao S, Gao X, Su Y, Qu P. Immunotherapy targeting myeloid-derived suppressor cells (MDSCs) in tumor microenvironment. Front Immunol. 2021;11:585214; 33613512 10.3389/fimmu.2020.585214 PMC7889583

[41] Chen S, Deng X, Xie C, Dong Q, Yang H. Near complete remission of a locally advanced giant melanoma of the vulva following hypo-fractionated radiotherapy and immune checkpoint inhibitors: a case report. Oncol Lett. 2022;24(6):458; 36380876 10.3892/ol.2022.13578 PMC9650599

